# Preparation and Application of Immobilized Surfactant-Modified PANi-CNT/TiO_2_ under Visible-Light Irradiation

**DOI:** 10.3390/ma10080877

**Published:** 2017-07-29

**Authors:** Ching Yuan, Chung-Hsuang Hung, Chung-Shin Yuan, Huei-Wen Li

**Affiliations:** 1 Department of Civil and Environmental Engineering, National University of Kaohsiung, No. 700, Kaohsiung University Rd., Nan-Tzu Dist., Kaohsiung 811, Taiwan; 2 Department of Safety, Health and Environmental Engineering, National Kaohsiung First University of Science and Technology, No. 1, University Rd., Yenchau Dist., Kaohsiung 824, Taiwan; jeremyh@ccms.nkfust.edu.tw; 3 Institute of Environmental Engineering, National Sun Yat-sen University, 70 Lienhai Rd., Kaohsiung 804, Taiwan; ycsngi@mail.nsysu.edu.tw; 4 Department of Civil and Environmental Engineering, National University of Kaohsiung, No. 700, Kaohsiung University Rd., Nan-Tzu Dist., Kaohsiung 811, Taiwan; soillab@nuk.edu.tw

**Keywords:** diethyl phthalate, immobilization, photo-catalysis, polyaniline, TiO_2_-based photocatalyst, visible light

## Abstract

Hydrothermally and sol-gel-synthesized immobilized surfactant-modified polyaniline-carbon nanotubes/TiO_2_ (PANi-CNT/TiO_2_) photocatalysts were prepared and their application in the degradation of diethyl phthalate (DEP) under visible light at 410 nm was investigated in this sturdy. To improve the dispersion of nanoparticles and the transfer of electrons, the TiO_2_ surface was modified with both sodium dodecyl sulfate (SDS) and functionalized carbon nanotubes (CNT-COOH and CNT-COCl). With the addition of PANi, which was increased from 1–5%, the adsorption edge of the prepared photocatalysts shifted to 442 nm. The SDS linked the PANi polymers to achieve a thickness of coating of the film of up to 314–400 nm and 1301–1600 nm for sol-gel hydrolysis and hydrothermally-synthesized photocatalysts, respectively. An appropriate film thickness would extend the transfer path of the electrons and inhibit the recombination of the electrons and the electron-holes. The photo-degradation performance of DEP by the hydrothermally-synthesized photocatalysts was better than those by sol-gel hydrolysis. The results revealed that the hydroxyl radicals were the key oxidant in the degradation of DEP using hydrothermally-synthesized PANi-CNT/TiO_2_ photocatalysts. The morphology and functional groups of the raw materials of photocatalysts were characterized and a comparison of photocatalytic activity with other TiO_2_-based photocatalysts was also provided.

## 1. Introduction

Phthalate esters (PAEs) are a variety of plasticizer that has been widely used in polyvinyl chloride plastics, styrene, cellulose film coating, cosmetics, adhesives, pulp and paper manufacturing [1,2]. PAEs are reported to be responsible for causing diseases such as liver cancer, breast cancers, genital lesions and testicular atrophy [3]. During product manufacturing and waste dumping, many phthalates-based chemicals are easily transferred to the environment because of their high water-solubility. Diethyl phthalate (DEP) is one of the most frequently identified esters in diverse environments, including surface marine water, freshwater, and sediments [4,5]. It was reported that the exposure of *Cyprinus carpio* to DEP in a dose as low as a 1/500th fraction of LC_50_ would result in metabolic changes and vitellogenin induction [6]. As PAEs are gradually accumulated in the environment and cause harmful effects to humans, effective treatment methods to attenuate the concentration of PAEs are needed.

Biodegradation, adsorption and oxidation processes have been attempted for the treatment of DEP. The biodegradation process [7,8] requires a longer time and produces a large amount of sludge. It has been reported that 83% of DEP was adsorbed by activated carbon at a pH of less than 7 [1]. Xu et al. [9] found that the adsorption capacity of DEP by two commercial resins was greater than that of activated carbon; however, these adsorbents need to be further treated, and the adsorption performance is significantly related to aqueous pH, which results in a higher treatment cost and lower treatment efficiency, respectively.

Among the oxidation processes, photo-catalysis has been recognized as one of the green technologies for the elimination of the recalcitrant contaminants in aqueous environments. This might be largely because this leads to the complete photo-destruction of various organic pollutants without chemical input or output and with no sludge residue [10,11]. Titanium dioxide (TiO_2_) has emerged as a photocatalyst leader for environmental decontamination, due to its high chemical stability, low cost and strong oxidizing power under ultraviolet irradiation [12,13,14]. However, the photocatalytic efficiency of TiO_2_ is restricted in terms of meeting practical needs under visible light irradiation because of the large intrinsic band gap (>3.2 eV) of TiO_2_ [11,15]. A variety of effective strategies have been adopted to enhance the photocatalytic efficiency of TiO_2_ materials. One of the most significant scientific and commercial advancements has been the development of visible light active TiO_2_ by chemical modifications in the TiO_2_ structure. Carbon nanotubes (CNTs) provide a large surface area and stabilize the charge separation by trapping the electrons transferred from TiO_2_; hence, they have been documented as good support materials for the enhancement of photo-induced catalysis, such as photo-catalysis and photo-electro-catalysis [16,17]. Consequently, CNTs have been used to upgrade TiO_2_ materials with a high photo-catalytic activity, as demonstrated in various publications [18,19,20]. The role of CNTs in TiO_2_-based photo-catalysis is as both as a stimulator, enhancing the injection of photo-excited electrons from TiO_2_, and as an electron sink, effectively separating electron-hole pairs; they also serve as a dispersing template to reduce the agglomeration of TiO_2_ nanoparticles [21,22,23].

Recently, conjugated polymers (CPs) have attracted considerable attention because of their excellent electron mobility and their distinguished ability to improve electrical conductivity, corrosion resistance, environmental stability, solar energy transfer and the photocatalytic activity of TiO_2_ [24,25,26,27]. They are suitable as stable photosensitizers to modify inorganic semiconductors such as TiO_2_ to fabricate optical, electronic, and photo-conversion devices. The photo-sensitizers greatly improved the charge separation efficiency and provided synergetic interactions between polymers and TiO_2_. Polyaniline (PANi) was one of the conjugated polymers applied to enhance the photocatalytic activity of TiO_2_ [28,29]. This was attributed to PANi acting as an efficient donor and transferrer of electrons generated from PANi to the TiO_2_ conduction band [30]. The addition of surfactants into sol-gel or hydrothermal solution as preparation for the photocatalysts could improve the hydrophilic characteristics of photocatalysts and result in a good dispersion of the photocatalysts. The commonly-used surfactants include sodium dodecyl sulfate (SDS), polyethylene glycol (PEG), Tween 80 and Tween 20 [31]. Yavuz and Gok [32] reported that the addition of SDS would reduce the degree of agglomeration of TiO_2_/PANi photocatalysts.

The development of immobilized photocatalysts is aimed at conquering the difficulty of recycling powder photocatalysts. The preparation methods for immobilized photocatalysts include sol-gel hydrolysis [33,34], hydrothermal synthesis [35], electrophoretic deposition [36], sputering [37] and electrospinning [38]. The adhesion strength is related to the service life of photocatalysts, which are varied with preparation methods. Among them, sol-gel hydrolysis and hydrothermal synthesis are the most common methods, because of their low cost and extensive application. The support material is critical for a successful immobilized photocatalyst. The important characteristics of support materials include the following: (1) a strong adherence between the photocatalyst and the support material; (2) no significant deterioration of the photo-catalytic activity by the preparation methods; (3) a high specific surface area; and (4) a strong adsorption affinity with the pollutants [39]. Due to their good thermal stability and light penetration, many types of glass were applied for photo-degradation in wastewater; i.e., glass plates, glass beads, glass tubes and glass rings [40,41].

In this study, we present a series of surfactant-modified PANi-CNT/TiO_2_ photocatalysts immobilized on a glass plate to degrade DEP. The PANi-CNT/TiO_2_ photocatalysts were prepared by co-doping with PANi and two functionalized CNT (CNT-COCl and CNT-COOH) onto TiO_2_ followed by sol-gel hydrolysis and hydrothermal synthesis. The comparison of the photo-catalytic activity of PANi-CNT/TiO_2_ photocatalysts prepared by two preparation methods was investigated in this study. The morphology and functional groups of the photo-catalytic base materials, as well as the characteristic absorption wavelength of PANi-CNT/TiO_2_, were also characterized.

## 2. Experimental Section

### 2.1. Reagents

Reagent-grade DEP, C_8_H_4_O_4_(C_2_H_5_)_2_, with a purity greater than 98%, was purchased from Sigma-Aldrich, St. Louis, MO, USA. Its chemical structure is similar to 17β estradiol, a natural hormone. As DEP is released into the environment, it can disturb hormone reactions in humans. DEP exhibits a higher affinity in the aqueous phase than in the soil phase, and the water solubility and the logarithm value of the octanol-water partition coefficient were 1080 mg/L and 2.7 (at 20 °C), respectively [42].

Titanium tetraisopropoxide (TTIP), the titanium source of TiO_2_, was obtained from Sigma-Aldrich, USA. Multi-wall carbon nanotubes (MWCNTs) were obtained from Centron Biochemistry Technology Co. Ltd., Taipei, Taiwan, and their properties were described in the previous study [19]. Sulfuric acid and nitric acid were purchased by Wako Pure Chemical Industries, Ltd., Osaka, Japan, which were used to functionalize the carboxyl group (–COOH) on the surface of the MWCNT. Isopropanol and thionyl chloride (SOCl_2_) were purchased from Merck Ltd., Kaohsiung, Taiwan, which were used to functionalize -COCl on the surface of the MWCNT. Dimethyl sulfoxide (DMSO) was purchased from Tedia Company Inc., Fairfield, OH, USA, which was used as the hydroxyl radical (OH•) scavenger. Sodium dihydrogen phosphate, 2,4-dinitrophenylhydrazine and formaldehyde were purchased from Merck Ltd., Taiwan, which were used to quantify the OH•.

### 2.2. Preparation of Immobilized SCPS, SGPS, HCPS, and HGPS Series Photocatalysts on a Glass Plate

The PANi-CNT/TiO_2_ photocatalysts were prepared with titanium isopropoxide (TTIP), two functionalized CNTs, PANi and SDS, followed by the sol-gel hydrolysis method and hydrothermal synthesis. The addition of SDS was to modify the characteristics of the photocatalyst from being hydrophobic to hydrophilic, which prevents the photocatalysts from agglomerating. Two functionalized CNTs [18], CNT-COOH and CNT-COCl, were used in this study, which have a more homogeneous adherence to base materials and a stronger reactivity than unfunctionalized CNTs. The PANi-TiO_2_/CNTs photocatalysts were prepared by sol-gel hydrolysis precipitation of titanium tetraisopropoxide (TTIP) onto functionalized CNTs, followed by calcination or hydrothermal treatment [19], which resulted in a di-crystalline TiO_2_ with anatase/rutile of 82/18. The PANi-CNT/TiO_2_ photocatalysts doped with CNT-COOH and CNT-COCl using sol-gel hydrolysis were named as SCPS and SGPS series photocatalysts, respectively. Similarly, the composite photocatalysts doped with CNT-COOH and CNT-COCl using hydrothermal synthesis were named as HCPS and HGPS series photocatalysts, respectively. The nomenclature of the composite photocatalysts was further based on the percentage of PANi and SDS; for example, SCP1S1 represents the SCPS series photocatalysts prepared with 1% of PANi and 1 cmc of SDS. We prepared a specific sol-gel solution (PANi/CNT-COOH/TiO_2_ and PANi/CNT-COCl/TiO_2_), the detailed preparation methods of which were described in the previous study [18], and immobilized 0.5 mL of this sol-gel solution on a glass plate (2.6 cm × 4 cm) by a spin-coater at 1000 rpm for 10 s and 5000 rpm for 50 s, respectively. The immobilized glass plate was moved into 80 °C oven for 1 h and cooled to room temperature. After the immobilized glass plate calcined in an oven at 550 °C for 1 h, the glass plate was immobilized with SCPS and SGPS series photocatalysts were ready for tests. For HCPS and HGPS series photocatalysts, the 12 pieces of glass plates (2.6 cm × 4 cm) were soaked into 100 mL of the above-mentioned sol-gel solution and were then moved into an autoclave to heat at 180 °C for 16 h. The immobilized HCPS and HGPS glass plates were washed with de-ionized water several times and dried at 80 °C for 1 h and then ready for use.

The chemical characterizations of the SCPS, SGPS, HCPS, and HGPS series photocatalysts were analyzed using scanning electron microscopy (SEM, Hitachi S4800, Tokyo, Japan), transmission electron microscopy (TEM, Hitachi S4800), Fourier transform infrared spectroscopy (FTIR, Perkin Elmer/Spectrum 100 Interior, Connecticut, NJ, USA) and a UV/Visible absorption spectrometer (JASCO V-760, Easton, MD, USA). The functional group on the surface of the photocatalysts was determined by FTIR. The charge characteristics of the photocatalysts in the aqueous phase were determined using a zeta potential meter. The characteristic absorption wavelength of the composite photocatalysts was analyzed using a UV-visible absorption spectrometer.

### 2.3. The Photocatalytic Degradation Tests

The photo reactor irradiated with 24 LED chips of 410 nm was established as shown in Hung et al. [18]. The power of each LED chip was 10 W and resulted in a light intensity of 40 mW/cm^2^. Each typical photo-catalytic experiment was conducted with four pieces of glass plates immobilized with SCPS, SGPS, HCPS and HGPS series photocatalysts, respectively. The total immersed area of the immobilized photocatalysts was 80.2 cm^2^. They were kept in suspension on a quartz cell containing 100 mL of 1 mg/L DEP at a pH of 7 for 120 min to investigate DEP degradation in this study. After dark adsorption for 20 min, LED light was used to irradiate and conduct photo-catalytic batch tests. An aqueous sample was taken every 20 min, and DEP was quantified using HPLC-UV (Waters 2695, Milford, MA, USA) with the Macherey-Nagel RP18 column (10 cm × 4.6 mm, 2.7 µm) at 224 nm [18]. A pseudo first-order kinetic model according to the Langmuir–Hinshewood kinetic mechanism [43] was applied to the photocatalytic degradation of DEP in this study. The apparent pseudo-first order rate constant, k_a_, will be quantified by photocatalytic degradation tests.

The photo-degradation efficiency is related to the amount of OH• in the system. It is difficult to directly quantify the OH• generated in the advanced oxidation system because of its high reaction rate. An alternative method was developed to use an effective scavenger to react with OH• and then quantify the production of products. The dimethyl sulfoxide (DMSO) acted as a scavenger in this study, which was successfully confirmed by Wu et al [44]. Lindsey and Tarr [45] reported that one mole of DMSO would react with 2.17 moles of OH• to produce 1 mole of formaldehyde. Hence, the generation rate of hydroxyl radical was equal to 2.17 times of formaldehyde generation. The concentration of formaldehyde was determined by HPLC-UV (Waters 2695, Milford, MA, USA) with Mightysil RP18 column (25 cm × 4.6 mm, 5 µm) at 370 nm. The isocratic elution was set at 70% methanol and 30% DI water with flow rate of 0.7 mL/min [46].

## 3. Results and Discussion

### 3.1. Characterization of PANI-CNT/TiO_2_ Photocatalysts

**Figure 1:**
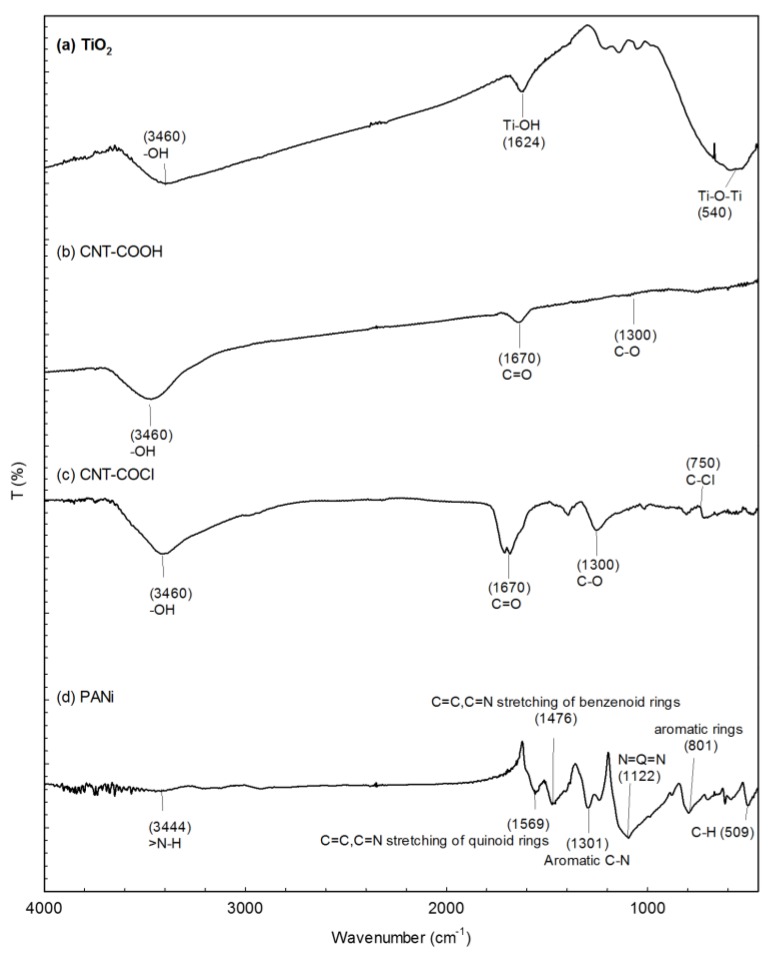
The Fourier transform infrared spectroscopy (FTIR) spectra of base materials for immobilized photocatalysts. (a) TiO_2_; (b) Carbon nanotubes (CNT)-COOH; (c) CNT-COCl; (d) Polyaniline (PANi).

All of the base materials for the PANI-CNT/TiO_2_ photocatalysts were prepared in this study. The exactly-defined properties of the base materials are the key factors to prepare good PANI-CNT/TiO_2_ photocatalysts. The FTIR patterns of these base materials of TiO_2_, CNT-COOH, and CNT-COCl are shown in Figure 1. The functional groups of Ti–O (3,400 1/cm), Ti–OH (1624 1/cm), and Ti-O-Ti (565 1/cm) were found in TiO_2_ (Figure 1a), which were the characteristic functional groups of TiO_2_ [47]. The functional groups of –OH (3460 1/cm), C=O (1670 1/cm), and C–O (1400 1/cm) were characterized in CNT-COOH (Figure 1b), which agreed with Chang et al. [48]. Other than –OH, C=O and C–O, the functional group of –Cl (750 1/cm) was also found in CNT-COCl (Figure 1c), which agreed with Inanaga et al. [49]. The results of the FTIR patterns confirmed that the carboxyl (–COOH) and acyl chloride (–OCl) group had been successfully grafted on the carbon nanotubes. The characteristic wavenumbers of PANi were characterized at 3444 1/cm (N–H), 1569 1/cm (C=C), 1476 1/cm (C=N), 1301 1/cm (C–N), and 1122 1/cm (N=Q=N), and 801 1/cm (aromatic rings) [22,29], which are also found in PANi prepared in this study (Figure 1d).

**Figure 2:**
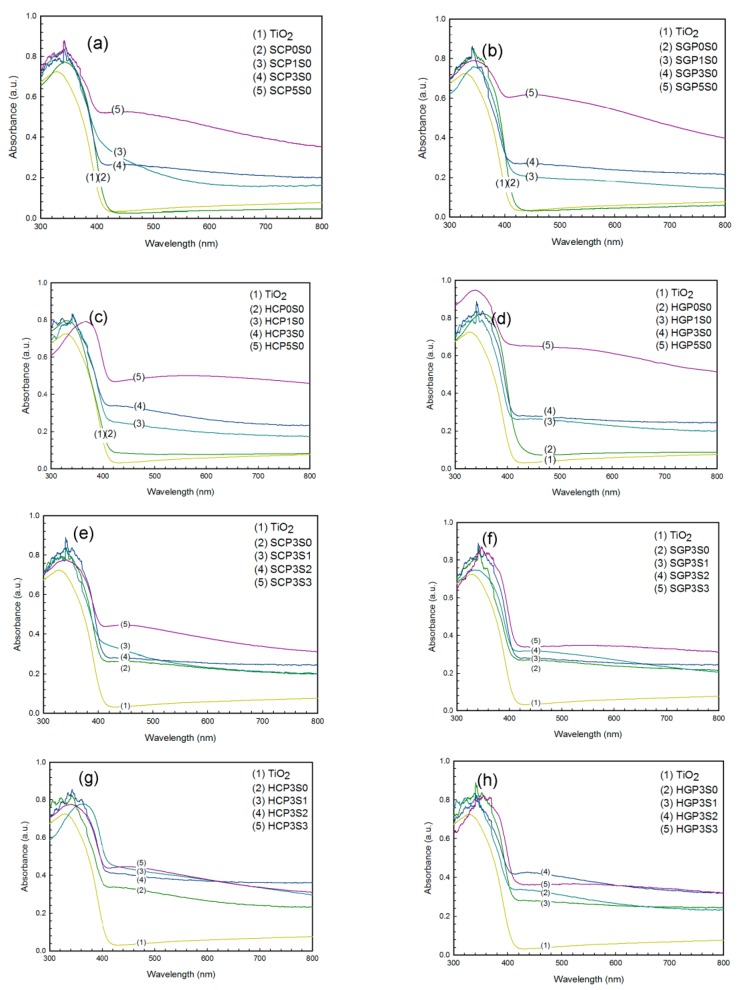
The UV-Vis absorption spectra of immobilized photocatalysts. (**a**–**d**) Effect of PANi on SCPS, SGPS, HCPS, and HGPS photocatalysts (no sodium dodecyl sulfate (SDS)); (**e**–**h**) Effect of SDS on SCP3S, SGP3S, HCP3S, and HGP3S photocatalysts.

The UV-Vis spectra of PANi-CNT/TiO_2_ photocatalysts are shown in Figure 2. The characteristic adsorption wavelength of TiO_2_ was determined at 387 nm. For CNT/TiO_2_ photocatalysts, the characteristic wavelength increased to 392–413 nm for the SCP0S0, SGP0S0, HCP0S0, and HGP0S0 photocatalysts, as shown in Figure 2a–d. This finding may be due to the functional group of C=O on CNT-COOH and CNT-COCl, shown in Figure 1b,c, being excited when adsorbing a light source greater than 400 nm. In addition to PANi increasing from 1–5%, the adsorption edge shifted from 421 to 423 nm, 415 to 440 nm, 403 to 417 nm, and 416 to 442 nm for the SCPS (Figure 2a), SGPS (Figure 2b), HCPS (Figure 2c), and HGPS (Figure 2d) series photocatalysts. The PANi could adsorb light with a wavelength greater than 490 nm [29], and consequently showed a distinguished red-shift effect of the adsorption edge for the PANi-CNT/TiO_2_ photocatalysts. The most distinguished red-shift of the adsorption edge was found for the SGPS and HGPS series photocatalysts.

The anionic surfactant, SDS, with both hydrophobic and hydrophilic characteristics, could link more PANi and photocatalysts together. The effects of the addition of SDS on the absorption edge of the photocatalysts are shown in Figure 2e–h. The absorption edge was determined to be at 422, 422, 426 and 418 nm for the SCP3S0, SGP3S0, HCP3S0 and HGP3S0 photocatalysts. As the addition of SDS increased from 1 cmc to 3 cmc (critical micelle concentration, 1 cmc = 8 mM), the red-shift of the adsorption edge was insignificant for the SCPS and SGPS photocatalysts (Figure 2e–f). This may be because the extra amount of PANi and CNT would be destroyed due to the high temperature in the sol-gel hydrolysis. A distinguished red-shift of the adsorption edge was found for the HCPS and HGPS series photocatalysts. With the addition of SDS up to 3 cmc, the adsorption edge shifted to 421 and 437 nm for HCP3S3 and HGP3S3, respectively (Figure 2g–h). The addition of SDS would increase the dispersion of PANi and CNT to result in more PANi coated on the TiO_2_.

### 3.2. The Morphology of PANi-CNT/TiO_2_ Photocatalysts

**Figure 3:**
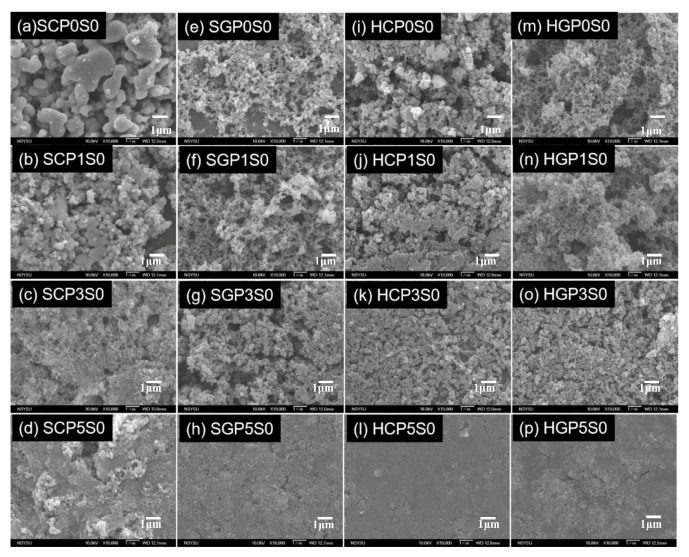
The scanning electron microscopy (SEM) images (10,000×) of immobilized PANi-CNT/TiO_2_ photocatalysts.

**Figure 4:**
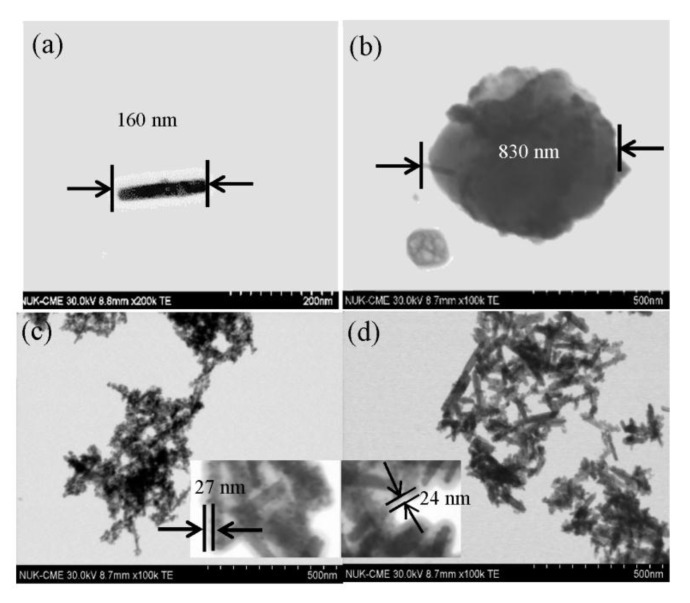
The transmission electron microscopy (TEM) images of (**a**) CNT; (**b**) PANi; (**c**) SGP3S3; and (**d**) HGP3S1.

**Figure 5:**
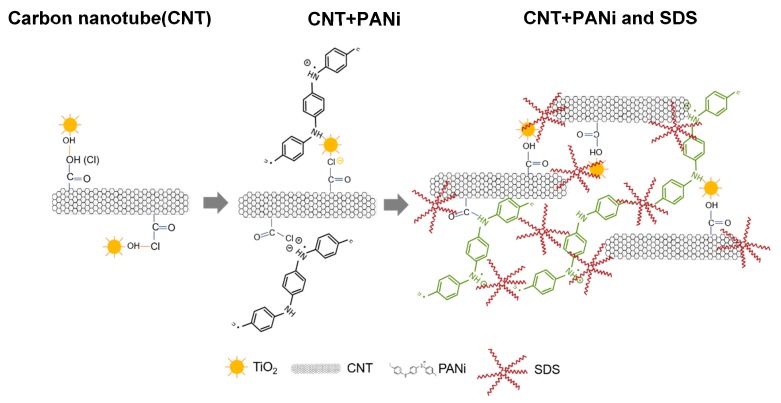
The proposed morphology of immobilized PANi-CNT/TiO_2_ photocatalysts.

Doping various amounts of functionalized CNT and PANi on TiO_2_ with either sol-gel or hydrothermal methods, four series photocatalysts of PANi-CNT/TiO_2_ were prepared in this study, for which the SEM images are summarized in Figure 3. The SEM micrograph revealed that the degree of agglomeration decreased in the direction from PANi of 1% to PANi of 5% for these four series of photocatalysts. This was in agreement with Radoičić, et al. [50], which also showed that the surface of TiO_2_ covered with more PANi would decrease the degree of agglomeration. Compared with SCPS/HCPS photocatalysts (Figure 3a–d,i–l), the results showed that a smoother surface on the SGPS/HGPS photocatalysts (Figure 3e–h,m–p) were found, which may be due to the binding strength between COCl and Ti–OH being stronger than that between COOH and Ti–OH [51,52]. Consequently, less agglomeration of TiO_2_ would be formed on the surface of PANi-CNT/TiO_2_ photocatalysts. As PANi increased up to 5%, a more compacted surface and less adsorption sites were found; however, the results showed that the porous characteristics of the HCPS/HGPS (Figure 3i–l/Figure 3m–p) photocatalysts were more significant than that on the SCPS/SGPS (Figure 3a–d/Figure 3e–h) photocatalysts. The PANi-CNT/TiO_2_ photocatalysts prepared with acyl chloride grafted CNT (CNT-COCl) by hydrothermal synthesis exhibited a porous surface and resulted in more adsorption sites after doping. The morphology of CNT, PANi, SGP3S3 and HGP3S1 visualized using a transmission electron microscope (TEM) are shown in Figure 4. Queiny et al. [53] reported that the morphology of CNT/PANi composites depends on the content of CNT and at a 2.7 wt % multiwall CNT loading, MWCNT/PANi are formed as individual nanofibers. The percentage of CNT for the prepared photocatalysts in this study was about 1%, so the morphology of SGP3S3 and HGP3S1 (Figure 4c,d) was similar to that reported by Queiny et al. [53]. The results in Figure 4 also indicated that the length of CNTs was approximately 165 nm, and PANi agglomerated as a cluster with a diameter of 830 nm. According to the size of CNTs and PANi, the CNT/TiO_2_ was partially encapsulated by PANi polymer. As shown in Figure 4c,d, it was obviously found that SGP3S3 and HGP3S1 were encapsulated with a PANi film approximately 20–30 nm thick. According to the images of the SEM and TEM, the morphology of the PANi-CNT/TiO_2_ photocatalysts are proposed in Figure 5. The functionalized CNTs provide a stronger binding with functional group of Ti-OH, especial for CNT-COCl, which resulted in a high conductivity for CNT/TiO_2_ photocatalysts. With the addition of PANi, its function group of NH would graft with CNT/TiO_2_ because of the free electron-holes of PANi attracting electrons of CNT/TiO_2_ [53]. This led the absorption edge of PANi-CNT/TuO_2_ shift to visible light. The addition of SDS would modify the hydrophobic characteristic of photocatalysts and link all the components together, which might result in more free transfer paths for electron migration.

**Figure 6:**
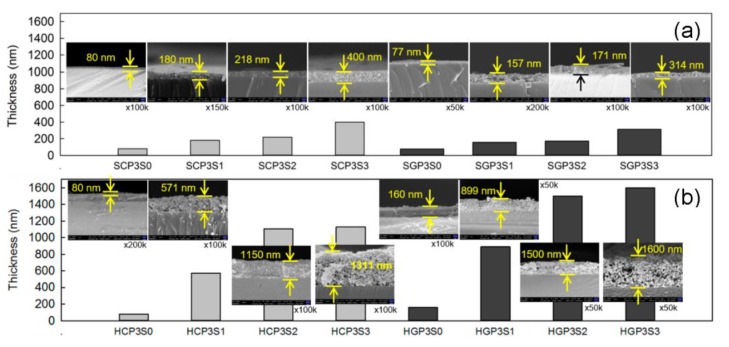
Coating film thickness of photocatalysts after prepared by (**a**) sol-gel hydrolysis; and (**b**) hydrothermal synthesis.

The addition of SDS into PANi-CNT/TiO_2_ photocatalysts would modify the hydrophobic characteristic of the photocatalysts and result in a thicker coating film. The thicker doping film will extend the transferring path of electrons, which favors photo-degradation performance; however, the thicker doping film will be easily detached. Hence, the optimal amount of SDS additive was the key factor for preparing good PANi-CNT/TiO_2_ photocatalysts. The results of the coating film thickness for the SCPS/SGPS photocatalysts and the HCPS/HGPS photocatalysts are shown in Figure 6a,b, respectively. The coating film thickness for the SCPS and SGPS photocatalysts was only 80 nm and 77 nm, respectively, with no addition of SDS. With the addition of 1–3 cmc of SDS, the coating film thickness increased to 180–400 nm for the SCPS photocatalysts and 157–314 nm for the SGPS photocatalysts, respectively. For the hydrothermal synthesis of photocatalysts with no addition of SDS, the coating film thickness was 188 nm and 160 nm for the HCPS and HGPS photocatalysts, respectively, which was 2.1–2.4 times greater than the SCPS/SGPS photocatalysts. With the addition of 1–3 cmc of SDS, the coating film thickness increased to 571–1105 nm for the HCPS photocatalysts and 889–1600 nm for the HGPS photocatalysts, which were 5.1–5.7 times greater than the coating film thickness of the SCPS/SGPS photocatalysts. This revealed that the PANi-CNT/TiO_2_ photocatalysts prepared with acyl chloride grafted CNT (CNT-COCl) by hydrothermal synthesis in this study exhibited a thicker coating film than SCPS/SGPS photocatalysts. Furthermore, the thickness of the coating film was positively correlated with SDS concentration. This may be largely due to the various preparation methods of the photocatalysts. The SCPS/SGPS photocatalysts were prepared using sol-gel hydrolysis at a high temperature and resulted in the extra dopant being removed by the centrifuge process. However, the HCPS/HGPS photocatalysts were prepared under a lower temperature, a higher pressure, and a longer aging time. The SDS plays an important role in linking between PANi polymers; this results in a thicker coating film, which agreed with Katoch et al. [54].

### 3.3. The Photodegradation of DEP by Sol-Gel-Synthesized PANi-CNT/TiO_2_ Photocatalysts

**Figure 7:**
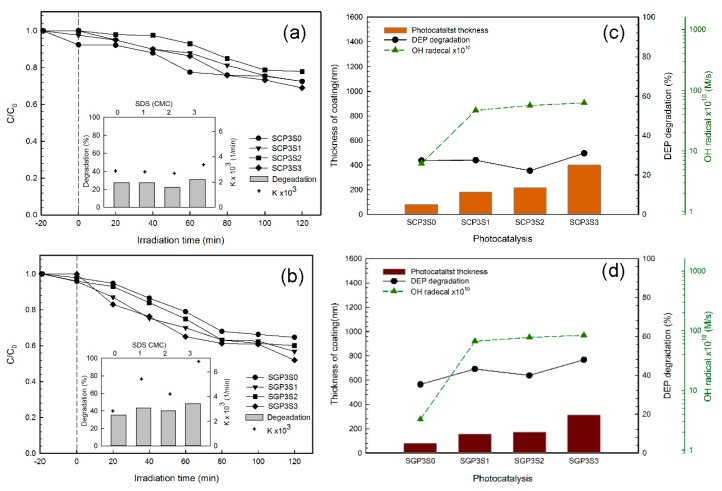
(**a**,**b**) Photodegradation of diethyl phthalate (DEP) by sol-gel hydrolysis photocatalysts ([DEP]_0_ = 1 mg/L; pH = 7; λ = 410 nm; I = 40 mW/cm^2^; Irradiation time = 120 min); (**c**,**d**) Photodegradation efficiency of DEP by sol-gel hydrolysis photocatalysts vs. hydroxyl radical generation and coating film thickness.

The photo-degradation of DEP by the SCPS and SGPS series photocatalysts is shown in Figure 7a,b, which obeyed the pseudo first-order kinetic model with an apparent pseudo-first order reaction rate constant (k_a_) of 2.7 × 10^−3^–3.3 × 10^−3^ 1/min and 4.1 × 10^−3^–6.8 × 10^−3^ 1/min as well as the regression constant (R^2^) of 0.881–0.892 and 0.935–0.955, respectively. The photo-degradation of DEP was 22.1–31.0% by the SCPS photocatalysts and 35.3–48.0% by the SGPS photocatalysts at a neutral pH under 410 nm irradiation for a 120 min irradiation. The results showed that the best photo-degradation activity of the SCPS and SGPS series photocatalysts was found at the addition of 3 cmc SDS. A positive correlation was found between DEP degradation and the apparent first-order rate constant of DEP (k_a_).

The hydroxyl radicals are a strong oxidizing species subject to the degradation of organic pollutants [28]. The graphs in Figure 7c,d illustrate the correlation between DEP degradation and the hydroxyl radical generation rate, as well as coating film thickness for the SCPS and SGPS series photocatalysts. A positive correlation was found between DEP degradation and coating film thickness. A thicker coating film would inhibit the recombination of electrons and electron-holes due to the extension of the electron’s transferring path, which resulted in the enhancement of photo-catalytic activity. Furthermore, an increase of film thickness also increases the amount of TiO_2_ and the area of the reaction field for photo-catalytic decomposition as judged from the porous structure in Figure 3. All these factors should contribute to the photo-catalytic decomposition. With the SDS concentration increased to 3 cmc, the hydroxyl radicals’ generation rate increased greatly, from 6.2 × 10^−10^ to 6.4 × 10^−9^ M/s for SCPS photocatalysts and from 3.3 × 10^−10^ to 8.3 × 10^−9^ M/s for SGPS photocatalysts. The DEP degradation was also positively correlated with the hydroxyl radical generation rate, except for SCP3S2 and SGP3S2. Jiménez et al. [40] reported that the defect sites easily accumulated electrons and promoted their attachment on O_2_ to produce powerful superoxide radicals (O_2_^−^•) in the photo-catalytic system. The superoxide radicals (O_2_^−^•) might be another key oxidant to degrade DEP for SCP3S2 and SGP3S2. In the investigated systems, the hydroxyl radical generation rate and the coating film thickness may be considered for the evaluation of the catalytic activity of photocatalysts.

### 3.4. The Photodegradation of DEP by Hydrothermally-Synthesized PANi-CNT/TiO_2_ Photocatalysts

**Figure 8:**
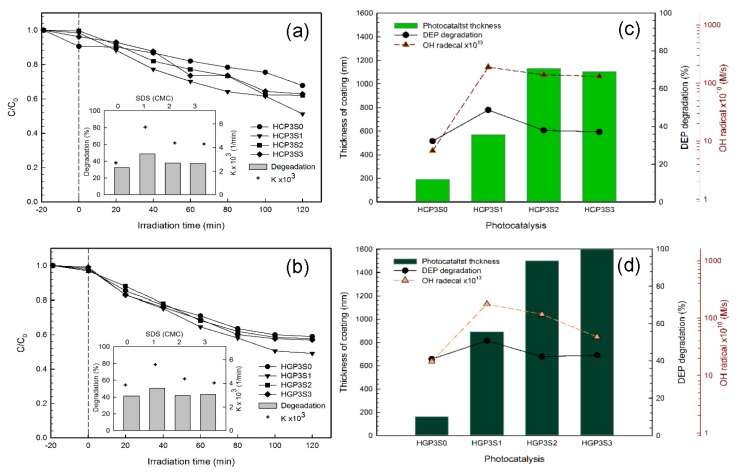
(**a**,**b**) Photo-degradation of DEP by hydrothermally-synthesized photocatalysts ([DEP]_0_ = 1 mg/L; pH = 7；λ = 410 nm; I = 40 mW/cm^2^; Irradiation time = 120 min); (**c**,**d**) Photo-degradation efficiency of DEP by hydrothermally-synthesized photocatalysts vs. hydroxyl radical generation and coating film thickness.

The photo-degradation of DEP by the HCPS and HGPS series photocatalysts is shown in Figure 8a,b, which obeyed the pseudo first-order kinetic model with an apparent pseudo-first order reaction rate constant (k_a_) of 2.7 × 10^−3^–5.7 × 10^−3^ 1/min and 3.9 × 10^−3^–5.6 × 10^−3^ 1/min as well as the regression constant (R^2^) of the 0.939–0.980, respectively. The photo-degradation of DEP was 32.2–48.7% by HCPS photocatalysts and 41.1–50.8% by HGPS photocatalysts at a neutral pH under 410 nm irradiation for 120 min. The results showed that the best degradation of DEP was found with the addition of 1 cmc of SDS. Organic residuals in the composite is an important factor of photo-catalytic activity. In the hydrothermal method, more SDS and PANi should remain in the composite, because the heating temperature was only 180 °C. For hydrothermal-treated photocatalysts, TiO_2_ in the composite may decompose not only DEP but also SDS and PANi. The organic residuals possibly decrease the decomposition rate of DEP. It could be a reason for the decrease of the photo-catalytic activity with a large amount of SDS. Besides this, photo-catalytic performance was dominated not only by the generation rate of reactive oxygen species (ROS, i.e., OH•, superoxide radicals) but also by the adsorption strength of chemicals on photocatalysts. The pH_ZPC_ of SCPS and SGPS series photocatalysts was in the range of 5.21–5.36 and 5.89–6.36. The pH_ZPC_ of HCPS and HGPS series photocatalysts was in the range of 5.41–7.10 and 5.99–7.31. Hence, for the degradation of DEP, the photo-catalytic activity of hydrothermally-synthesized photocatalysts was better compared to the sol-gel hydrolysis photocatalysts at a neutral pH.

The graphs in Figure 8c,d illustrate the correlation between DEP degradation and the hydroxyl radical generation rate, as well as coating film thickness for the HCPS and HGPS series photocatalysts. A positive correlation was found between the degradation of DEP and the hydroxyl radical generation rate. It indicated that the hydroxyl radical was the key oxidant to degrade DEP, which was different from the sol-gel hydrolysis photocatalysts. The coating film thickness may be another key factor that affects the degradation of DEP. The coating film thickness was greater than 1000 nm for the HCP3S2 and HCP3S3 photocatalysts, which was up to 1.8 times and 2.0 times greater than that of HCP3S1 and HGP3S2, respectively. The thicker coating film may be easily detached in the photocatalytic system and results in less generated hydroxyl radicals, as shown in Figure 8c,d, which was also agrees with Shan et al. [41]. It can be inferred that the degradation of DEP was dominated by the coating film thickness of the immobilized photocatalysts.

### 3.5. Characteristics and Photocatalytic Activity of PANi-CNT/TiO_2_ Photocatalysts

**Figure 9:**
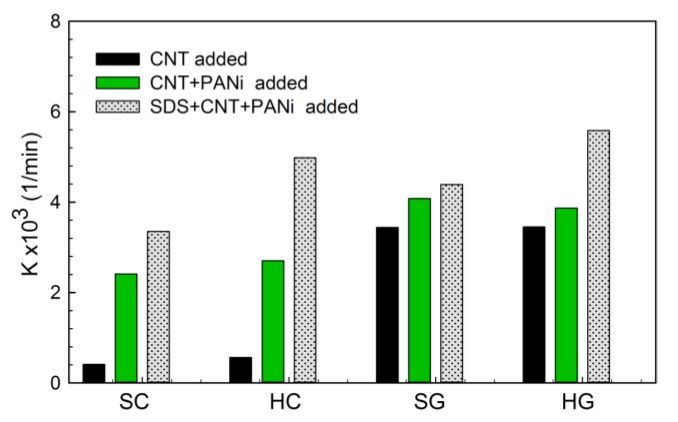
Contribution of composite materials to photocatalytic activity. SDS: sodium dodecyl sulfate.

The contribution of CNT, PANi and SDS to the photo-catalytic activity of surfactant modified PANi-CNT/TiO_2_ prepared in this study irradiated with 410 nm is shown in Figure 9. The results showed that, with addition of PANi, the reaction rate constants of SC and HC photocatalysts increased from 0.41 × 10^−3^ 1/min to 2.4 × 10^−3^ 1/min and from 0.57 × 10^−3^ 1/min to 2.70 × 10^−3^ 1/min, respectively, which showed that 4.8–5.9 times of photocatalytic activity were enhanced. For SG and HG photocatalysts, only 1.1–1.2 times of reaction rates were increased. With the addition of SDS, 1.4–1.8 time and 1.1–1.4 time of photocatalytic ativity was increased for hydrothermally-synthersized photocatalysts (HC and HG) and sol-gel-synthesized photocatalysts (SC and SG), respectively. For sol-gel-synthesized photocatalysts, both PANi and SDS were important to enhance the photo-catalytic acticity, whereas, for hydrotermally-synthesized photocatalysts, the contribution of SDS to photocatalytic activity was more critical.

**Figure 10:**
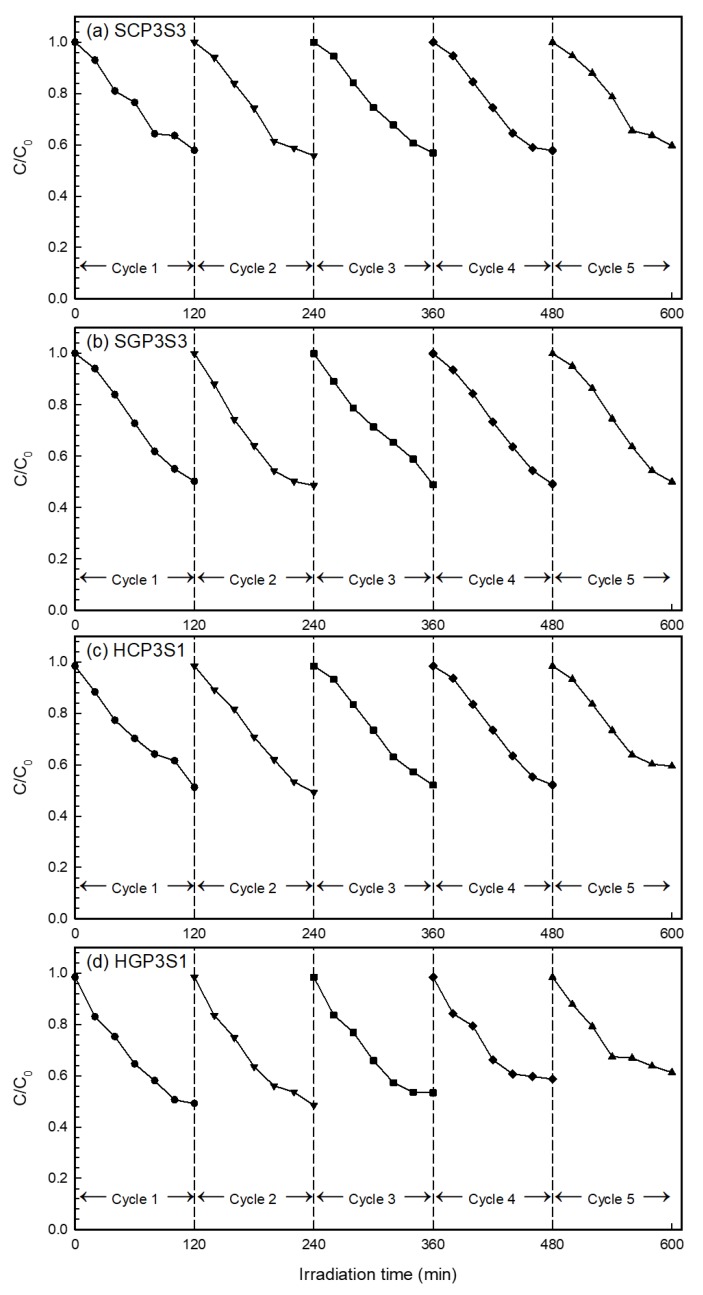
Cyclic photocatalytic capability of immobilized photocatalysts. (a) SCP3S3; (b) SGP3S3; (c) HCP3S1; and (d) HGP3S1.

The cyclic runs of the photo-degradation of DEP with immobilized surfactant-modified PANi/CNT/TiO_2_ photocatalysts were conducted to evaluate their photo-catalytic stability and recyclability. The four investigated photocatalysts were repeatedly tested up to five times; the results of which are shown in Figure 10. The five-cycle repeated photo-catalytic degradation efficiency of DEP for SCP3S3 was 42.1%, 44.2%, 43.2%, 42.2% and 40.3%, respectively. This showed that the degradation efficiency for SCP3S3 was only 1.8% less after being used five times. For SGP3S3, the five-cycle repeated photo-catalytic degradation efficiency of DEP was 49.8%, 51.5%, 51.1%,50.9% and 50.0%, respectively, which showed that the degradation efficiency of DEP at the fifth cycle was similar to that at the first cycle. For HCP3S1, the five-cycle repeated photo-catalytic degradation efficiency of DEP was 48.7%, 50.7%, 47.9%, 47.9% and 40.5%. This showed the degradation efficiency for HCP3S3 was 8.3% less after being used for five cycles. For HGP3S1, the five-cycle repeated photo-catalytic degradation efficiency of DEP was 50.8%, 51.5%, 46.6%, 41.3% and 38.7%. Compared to the other three photocatalysts, the degradation efficiency for HGP3S3 after being used for five cycles was significantly decreased. The results in Figure 10 illustrated that the photo-catalytic performance at the first cycle for all photocatalysts was not superior to those of the other subsequent uses. This agreed quite well with Hung et al. [18] and Sriwong et al. [55], who reported that the surface of the photocatalyst was covered with trace impurities when freshly prepared. The photo-catalytic activity of all immobilized photocatalysts were stable for at least five cycles.

Table 1 summarizes the photo-catalytic activity of TiO_2_-based photocatalysts. The photo-catalytic activity is closely related to many factors, including the anatase/rutile ratio of TiO_2_, chemicals/concentration, light source/intensity, dopant and preparation methods. The results showed that the addition of PANi into TiO_2_-bsed photocatalysts made less of a difference to photo-catalytic activity under UV and visible light irradiation [29,56,57,58]. Nevertheless, a better photo-catalytic performance of TiO_2_-based photocatalysts without PANi addition was found when irradiated with UV light [59,60]. The reaction rate constants of PANi-CNT/TiO_2_ photocatalysts (1.8 × 10^−3^–5.7 × 10^-3^ 1/min) were similar or even higher than most of those TiO_2_-based photocatalysts listed in Table 1. Radoičić et al. [50] reported a higher rate constant (1.8 × 10^−2^–7.8 × 10^−2^ 1/min) of PANi/TiO_2_ photocatalysts under simulated solar light than that found in this study. This might be largely because of a higher rutile phase (38%) than the PANi-CNT/TiO_2_ prepared in this study. The superiorities of the PANi-CNT/TiO_2_ photocatalysts were revealed because of the recalcitrant characteristics of diethyl phthalate. It can be expected that the development of PANi-CNT/TiO_2_ photocatalysts with a higher percentage of their rutile phase will enhance the photo-catalytic activity in the visible spectrum.

Compared with other TiO_2_-based photocatalysts listed in Table 1, three advantages of PANi-CNT/TiO_2_ photocatalysts prepared in this study were found. The first was that the di-crystalline phase of TiO_2_, the anatase/rutile phase of 82/18, inhibits the recombination of the electron and the electron-hole. Second, the addition of SDS led to a thicker doping film and extended the transfer path of electrons. Third, the characteristics of the long wavelength absorption of PANi shifted the absorbing edge of the PANi-CNT/TiO_2_ photocatalysts to visible light, which enhanced the photocatalytic activity in the visible spectrum.

## 4. Conclusions

A novel CNT/TiO_2_ photocatalyst modified with PANi and SDS immobilized on a glass plate prepared with sol-gel hydrolysis and hydrothermal synthesis was investigated to evaluate the photo-catalytic performance of DEP under visible light in this work. The properties of highly conducting and absorbing visible light for PANi enable both the easy release of electrons and the shift of the characteristic absorption edge of photocatalysts to the visible spectrum. The anionic surfactant, SDS, increases the dispersion of PANi and results in more PANi homogeneously coated on the TiO_2_. The important conclusions in this work are as follows:

1. Owing to PANi with the characteristics of absorbing visible light, the adsorption edge of surfactant- modified PANi-CNT/TiO_2_ photocatalysts shifted up to 442 nm when PANi addition was increased up to 5%. The most distinguished red-shift of the adsorption edge was found for the SGPS and HGPS series photocatalysts.
2. The anionic surfactant, SDS, plays an important role in determining a link between the PANi polymers and the coating film up to 314–400 nm and 1301–1600 nm for so-gel hydrolysis and hydrothermally-synthesized photocatalysts, respectively. An appropriate coating film thickness would extend the transfer path of electrons and inhibit the recombination of electrons and electron-holes. Hence, the coating film thickness of immobilized photocatalysts may be considered for the evaluation of the photo-catalytic activity.
3. The degradation of DEP by both sol-gel synthesized PANi-CNT/TiO_2_ photocatalysts and hydrothermally-synthesized PANi-CNT/TiO_2_ photocatalysts obeyed the pseudo first-order kinetic model. The photo-degradation of DEP by hydrothermally synthesized PANi-CNT/TiO_2_ photocatalysts under 410-nm irradiation was better than that by sol-gel hydrolysis PANi-CNT/TiO_2_ photocatalysts. This may be due to the lower fraction of PANi and CNT which was found in the sol-gel hydrolysis photocatalysts due to their high-temperature preparation.
4. The results of the hydroxyl radical quantification revealed that the hydroxyl radicals were the key oxidant for the degradation DEP for the hydrothermally-synthesized PANi-CNT/TiO_2_ photocatalysts, but this was not true for the sol-gel hydrolysis photocatalysts. Further investigation is needed for the generation of any other radicals, such as superoxide radicals (O_2_^−^•), in the sol-gel hydrolysis photocatalysts’ system, in order to clarify the degradation mechanisms.
5. For sol-gel-synthesized photocatalysts, both PANi and SDS were important to enhance the photo-catalytic acticity; whereas, for hydrotermally-synthesized photocatalysts, the contribution of SDS to photocatalytic activity was more critical. Their photocatalytic activities were stable for at least five cycles. The surface of the photocatalyst was covered with trace impurities when it was freshly prepared; hence, the photo-catalytic performance at the first cycle for all immobilized photocatalysts was not superior to those of the other subsequent uses.

## Figures and Tables

**Table 1:** Comparative photocatalytic performance of TiO_2_-based photocatalysts.

References	TiO_2_ Source; Preparation Method; A/R *	Dopant	Polymer; Concentration	Surfactant	Light Source; Intensity/Power	Chemicals; Concentration	Reaction Rate Constant (1/min)
Li et al. [56]	tetrabutyl titanate ; sol-gel hydrolysis; 100/0	-	PANi; 1.6–3.2 wt %	-	λ > 400 nm; 300 W	phenol; 50 mg/L	2.7 × 10^−3^–3.1 × 10^−3^
Huang et al. [57]	Fe_3_O_4_/SiO_2_/TiO_2_(TBT); sol–gel hydrolysis; 100/0	Fe_3_O_4_/SiO_2_	PANi; 2–4.2 wt %	-	420 nm; not mentioned	Methyl blue; 10 mg/L	9.5 × 10^−4^–1.3 × 10^−3^
Radoičić et al. [29]	TiCl_4_; sol-gel hydrolysis; 100/0	-	PANi; 0.5–1.5 wt %	-	UVB (280~315 nm); 3 W UVA (315~400 nm); 13.6W	Methyl blue; 10^−5^ M Rhodamine B; 10^−5^ M	3.2 × 10^−2^–1.2 × 10^−3^; 9.4 × 10^−4^–5.4 × 10^−3^
Yang et al. [58]	Ti foil; - ; not mentioned	Cr	PANi; not mentioned	-	Λ = 253.7 nm; 15 W	p-Nitrophenol; not mentioned	6.3×10^−3^–10.3×10^−3^
Nourbakhsh et al. [59]	TTIP; sol-gel hydrolysis; 50/50	Cu: 2–13%, CNT: 11%	-	-	UV 325 nm; 15 W	Methyl orange; not mentioned	TiO_2_/CNT: 4×10^−2^; TiO_2_/CNT–7%Cu: 5 × 10^−2^; TiO_2_/CNT–10%Cu: 1.3 × 10^−1^; TiO_2_/CNT–20%Cu: 2.3 × 10^−1^;
Zouzelka et al. [60]	TTIP; hydrothermal; 100/0	CNT	-	-	UV 365 nm; 11 W	4-Chlorophenol; 0.1 mM	3.5 × 10^−3^–3.6 × 10^−3^
Radoičić et al. [50]	TiCl_4_; chemical oxidative polymerization; 62/38	-	[TiO_2_]/PANi] = 20–80 (mole ratio)	-	Simulated solar light	Methylene blue Rhodamine B	1.8×10^-2^–7.8 ×10^-2^ 1.7×10^-2^–3.3 ×. 10^-2^
Li et al. [61]	TiCl_4_; sol-gel hydrolysis; 100/0	-	PANi: 10–20 wt %	SDS: 1%	UV 325 nm; 0.5–1 mW/cm^2^	Methyl blue; 10^-5^ M	1.4×10^-3^–1.5 ×10^-3^
This study	TTIP; sol-gel hydrolysis; 82/18	CNT: 1%	PANi: 1–3 wt %	SDS: 1-3 cmc	410 nm; 40 mW/cm^2^	Diethyl phthalate; 1 mg/L	2.8×10^-3^– 6.8×10^-3^
This study	TTIP; hydrothermal synthesis; 82/18	CNT: 1%	PANi: 1-3 wt %	SDS: 1-3 cmc	410 nm; 40 mW/cm^2^	Diethyl phthalate; 1 mg/L	1.8×10^-3^–5.7×10^-3^

* A/R: crystalline phase ratio of anatase /rutile.

## References

[1] Venkata Mohan S., Shailaja S., Rama Krishna M., Sarma P.N. (2007). Adsorptive removal of phthalate ester (Di-ethyl phthalate) from aqueous phase by activated carbon: a kinetic study. J. Hazard. Mater..

[2] Wu M.H., Liu N., Xu G., Ma J., Tang L., Wang L., Fu H.Y. (2011). Kinetics and mechanisms studies on dimethyl phthalate degradation in aqueous solutions by pulse radiolysis and electron beam radiolysis. Radiat. Phys. Chem..

[3] Kaneco S., Rahman M.A., Suzuki T., Katsumata H., Ohta K. (2004). Optimization of solar photocatalytic degradation conditions of bisphenol A in water using titanium dioxide. J. Photochem. Photobiol.

[4] Jing Y., Li L.S., Zhang Q.Y., Lu P., Liu P.H., Lu X.H. (2011). Photocatalytic ozonation of dimethyl phthalate with TiO_2_ prepared by a hydrothermal method. J. Hazard. Mater..

[5] Cinelli G., Avino P., Notardonato I., Centola A., Russo M.V. (2014). Study of XAD-2 adsorbent for the enrichment of trace levels of phthalate esters in hydroalcoholic food beverages and analysis by gas chromatography coupled with flame ionization and ion-trap mass spectrometry detectors. Food Chem..

[6] Barse A.V., Chakrabarti T., Ghosh T.K., Pal A.K., Jadhao S.B. (2007). Endocrine disruption and metabolic changes following exposure of *Cyprinus. carpio* to diethyl phthalate. Pestic. Biochem. Physiol..

[7] Na S., Jinhua C., Cui M., Khim J. (2012). Sonophotolytic diethyl phthalate (DEP) degradation with UVC or VUV irradiation. Ultrason. Sonochem..

[8] Roslev P., Vorkamp K., Aarup J., Frederiksen K., Nielsen P.H. (2007). Degradation of phthalate esters in an activated sludge wastewater treatment plant. Water Res..

[9] Xu Z., Zhang W., Pan B., Hong C., Lv L., Zhang Q., Pan B., Zhang Q. (2008). Application of the Polanyi potential theory to phthalates adsorption from aqueous solution with hyper-cross-linked polymer resins. J. Colloid Interface Sci..

[10] Vimonses V., Jin B., Chow C.W.K., Saint C. (2010). An adsorption–photocatalysis hybrid process using multi-functional-nanoporous materials for wastewater reclamation. Water Res..

[11] Liu L., Bai H., Liu J., Sun D.D. (2013). Multifunctional graphene oxide-TiO_2_-Ag nanocomposites for high performance water disinfection and decontamination under solar irradiation. J. Hazard. Mater..

[12] Oros-Ruiz S., Zanella R., Prado B. (2013). Photocatalytic degradation of trimethoprim by metallic nanoparticles supported on TiO_2_-P25. J. Hazard. Mater..

[13] Asiltürk M., Şener Ş. (2012). TiO_2_-activated carbon photocatalysts: Preparation, characterization and photocatalytic activities. Chem. Eng. J..

[14] Singh S., Mahalingam H., Singh P.K. (2013). Polymer-supported titanium dioxide photocatalysts for environmental remediation: A review. Appl. Catal., A-Gen..

[15] Fang J., Xu L., Zhang Z., Yuan Y., Cao S., Wang Z., Yin L., Liao Y., Xue C. (2013). Au@TiO_2_–CdS Ternary Nanostructures for Efficient Visible-Light-Driven Hydrogen Generation. ACS Appl. Mater. Interfaces.

[16] Zhao Y., Pan F., Li H., Zhao D., Liu L., Xu G.Q., Chen W. (2013). Uniform mesoporous anatase–brookite biphase TiO_2_ hollow spheres with high crystallinity via ostwald ripening. J. Phys. Chem..

[17] Byrappa K., Dayananda A.S., Sajan C.P., Basavalingu B., Shayan M.B., Soga K., Yoshimura M. (2008). Hydrothermal preparation of ZnO:CNT and TiO_2_:CNT composites and their photocatalytic applications. J. Mater. Sci..

[18] Hung C.H., Yuan C., Li H.W. (2017). Photodegradation of diethyl phthalate with PANi-CNT/TiO_2_ immobilized on glass plate irradiated with visible light and simulated sunlight—effect of synthesized method and pH. J. Hazard. Mater..

[19] Hung C.H., Chuang B.C., Lien H.L., Yuan C., Doong R.A., Sharma Virender K., Kim H. (2013). Photocatalytic Degradation of Bisphenol A Using TiO_2_/CNTs Nanocomposites under UV Irradiation. Interactions of Nanomaterials with Emerging Environmental Contaminants.

[20] Zhang K., Zhang F.J., Chen M.L., Oh W.C. (2011). Comparison of catalytic activities for photocatalytic and sonocatalytic degradation of methylene blue in present of anatase TiO_2_–CNT catalysts. Ultrason. Sonochem..

[21] Miranda S.M., Romanos G.E., Likodimos V., Marques R.R.N., Favvas E.P., Katsaros F.K., Stefanopoulos K.L., Vilar V.J.P., Faria J.L., Falaras P., Silva A.M.T. (2014). Pore structure, interface properties and photocatalytic efficiency of hydration/dehydration derived TiO_2_/CNT composites. Appl. Catal. B Environ..

[22] Silva C.G., Faria J.L. (2010). Photocatalytic oxidation of benzene derivatives in aqueous suspensions: Synergic effect induced by the introduction of carbon nanotubes in a TiO_2_ matrix. Appl. Catal. B Environ..

[23] Xu Y.J., Zhuang Y., Fu X. (2010). New Insight for Enhanced Photocatalytic Activity of TiO_2_ by Doping Carbon Nanotubes: A Case Study on Degradation of Benzene and Methyl Orange. J. Phys. Chem. C.

[24] Reddy K.R., Sin B.C., Ryu K.S., Kim J.C., Chung H., Lee Y. (2009). Conducting polymer functionalized multi-walled carbon nanotubes with noble metal nanoparticles: Synthesis, morphological characteristics and electrical properties. Synth. Met..

[25] Reddy K.R., Nakata K., Ochiai T., Murakami T., Tryk D.A., Fujishima A. (2010). Nanofibrous TiO_2_-Core/Conjugated Polymer-Sheath Composites: Synthesis, Structural Properties and Photocatalytic Activity. J. Nanosci. Nanotechnol..

[26] Park O.K., Hahm M.G., Lee S., Joh H.I., Na S.I., Vajtai R., Lee J.H., Ku B.C., Ajayan P.M. (2012). In situ synthesis of thermochemically reduced graphene oxide conducting nanocomposites. Nano. Lett..

[27] Zhang H., Zong R., Zhu Y. (2009). Photocorrosion Inhibition and Photoactivity Enhancement for Zinc Oxide via Hybridization with Monolayer Polyaniline. J. Phys. Chem. C..

[28] Lin C.J., Liou Y.H., Zhang Y., Chen C.L., Dong C.L., Chen S.Y., Stucky G.D. (2012). Mesoporous Fe-doped TiO_2_ sub-microspheres with enhanced photocatalytic activity under visible light illumination. Appl. Catal. B Environ..

[29] Radoičić M., Šaponjić Z., Janković I.A., Ćirić-Marjanović G., Ahrenkiel S.P., Čomor M.I. (2013). Improvements to the photocatalytic efficiency of polyaniline modified TiO_2_ nanoparticles. Appl. Catal. B Environ..

[30] Reddy K.R., Hassan M., Gomes V.G. (2015). Hybrid nanostructures based on titanium dioxide for enhanced photocatalysis. Appl. Catal. A Gen..

[31] Chen K., Li J., Wang W., Zhang Y., Wang X., Su H. (2012). Effects of surfactants on microstructure and photocatalytic activity of TiO_2_ nanoparticles prepared by the hydrothermal method. Mater. Sci. Semicond. Process..

[32] Yavuz A.G., Gok A. (2007). Preparation of TiO_2_/PANi composites in the presence of surfactants and investigation of electrical properties. Synth. Met..

[33] Lesch A., Cortés-Salazar F., Prudent M., Delobel J., Rastgar S., Lion N., Tissot J.D., Tacchini P., Girault H.H. (2014). Large scale inkjet-printing of carbon nanotubes electrodes for antioxidant assays in blood bags. J. Electroanal. Chem..

[34] Subramanian M., Vijayalakshmi S., Venkataraj S., Jayavel R. (2008). Effect of cobalt doping on the structural and optical properties of TiO_2_ films prepared by sol–gel process. Thin Solid Films.

[35] Guo W., Liu X., Huo P., Gao X., Wu D., Lu Z., Yan Y. (2012). Hydrothermal synthesis spherical TiO_2_ and its photo-degradation property on salicylic acid. Appl. Surf. Sci..

[36] Dunlop P.S.M., McMurray T.A., Hamilton J.W.J., Byrne J.A. (2008). Photocatalytic inactivation of Clostridium perfringens spores on TiO_2_ electrodes. J. Photochem. Photobiol. A.

[37] Raja P., Nadtochenko V., Klehm U., Kiwi J. (2008). Structure and performance of a novel TiO_2_-phosphonate composite photocatalyst. Appl. Catal. B Environ..

[38] Choi K.I., Lee S.H., Park J.Y., Choi D.Y., Hwang C.H., Lee I.H., Chang M.H. (2013). Fabrication and characterization of hollow TiO_2_ fibers by microemulsion electrospinning for photocatalytic reactions. Mater. Lett..

[39] Vaiano V., Sacco O., Sannino D., Ciambelli P. (2015). Nanostructured N-doped TiO_2_ coated on glass spheres for the photocatalytic removal of organic dyes under UV or visible light irradiation. Appl. Catal. B Environ..

[40] Jiménez M., Ignacio Maldonado M., Rodríguez E.M., Hernández-Ramírez A., Saggioro E., Carra I., Sánchez Pérez J.A. (2015). Supported TiO_2_ solar photocatalysis at semi-pilot scale: degradation of pesticides found in citrus processing industry wastewater, reactivity and influence of photogenerated species. J. Chem. Technol. Biotechnol..

[41] Shan A.Y., Ghazi T.I.M., Rashid S.A. (2010). Immobilisation of titanium dioxide onto supporting materials in heterogeneous photocatalysis: A review. Appl. Catal. A Gen..

[42] Abdel daiem M.M., Rivera-Utrilla J., Ocampo-Perez R., Mendez-Diaz J.D., Sanchez-Polo M. (2012). Environmental impact of phthalic acid esters and their removal from water and sediments by different technologies--a review. J. Environ. Manag..

[43] Wang H., Wang H.L., Jiang W.F. (2009). Solar photocatalytic degradation of 2,6-dinitro-p-cresol (DNPC) using multi-walled carbon nanotubes (MWCNTs)–TiO_2_ composite photocatalysts. Chemosphere.

[44] Wu K.R., Hung C.H. (2009). Characterization of N,C-codoped TiO_2_ films prepared by reactive DC magnetron sputtering. Appl. Surf. Sci..

[45] Lindsey M.E., Tarr M.A. (2000). Quantitation of hydroxyl radical during fenton oxidation following a single addition of iron and peroxide. Chemosphere.

[46] Lee Y., Lee C., Yoon J. (2004). Kinetics and mechanisms of DMSO (dimethylsulfoxide) degradation by UV/H_2_O_2_ process. Water Res..

[47] Subramanian E., Subbulakshmi S., Murugan C. (2014). Inter-relationship between nanostructures of conducting polyaniline and the photocatalytic methylene blue dye degradation efficiencies of its hybrid composites with anatase TiO_2_. Mater. Res. Bull..

[48] Chang C.F., Man C.Y. (2011). Titania-coated magnetic composites as photocatalysts for phthalate photodegradation. Ind. Eng. Chem. Res..

[49] Inanaga J., Hirata K., Saeki H., Katsuki T., Yamaguchi M. (1979). A Rapid Esterification by Means of Mixed Anhydride and Its Application to Large-ring Lactonization. Bull. Chem. Soc. Jpn..

[50] Radoičić M., Ćirić-Marjanović G., Spasojević V., Ahrenkiel P., Mitrić M., Novaković T., Šaponjić Z. (2017). Superior photocatalytic properties of carbonized PANI/TiO_2_ nanocomposites. Appl. Catal. B Environ..

[51] Massoumi B., Jaymand M., Samadi R., Entezami A. (2014). In situ chemical oxidative graft polymerization of thiophene derivatives from multi-walled carbon nanotubes. J. Polym. Res..

[52] Sahoo N.G., Cheng H.K.F., Li L., Chan S.H., Judeh Z., Zhao J. (2009). Specific Functionalization of Carbon Nanotubes for Advanced Polymer Nanocomposites. Adv. Funct. Mater..

[53] Oueiny C., Berlioz S., Perrin F.X. (2014). Carbon nanotube–polyaniline composites. Prog. Polym. Sci..

[54] Katoch A., Burkhart M., Hwang T., Kim S.S. (2012). Synthesis of polyaniline/TiO_2_ hybrid nanoplates via a sot-gel chemical method. Chem. Eng. J..

[55] Sriwong C., Wongnawa S., Patarapaiboolchai O. (2012). Rubber sheet strewn with TiO_2_ particles: Photocatalytic activity and recyclability. J. Environ. Sci..

[56] Li X., Wang D., Cheng G., Luo Q., An J., Wang Y. (2008). Preparation of polyaniline-modified TiO_2_ nanoparticles and their photocatalytic activity under visible light illumination. Appl. Catal. B Environ..

[57] Huang X., Wang G., Yang M., Guo W., Gao H. (2011). Synthesis of polyaniline-modified Fe_3_O_4_/SiO_2_/TiO_2_ composite microspheres and their photocatalytic application. Mater. Lett..

[58] Yang K., Pu W., Tan Y., Zhang M., Yang C., Zhang J. (2014). Enhanced photoelectrocatalytic activity of Cr-doped TiO_2_ nanotubes modified with polyaniline. Mater. Sci. Semicond. Process..

[59] Nourbakhsh A., Abbaspour S., Masood M., Mirsattari S.N., Vahedi A., Mackenzie K.J.D. (2016). Photocatalytic properties of mesoporous TiO_2_ nanocomposites modified with carbon nanotubes and copper. Ceram. Int..

[60] Zouzelka R., Kusumawati Y., Remzova M., Rathousky J., Pauporte T. (2016). Photocatalytic activity of porous multiwalled carbon nanotube-TiO_2_ composite layers for pollutant degradation. J. Hazard. Mater..

[61] Li Y., Yu Y., Wu L., Zhi J. (2013). Processable polyaniline/titania nanocomposites with good photocatalytic and conductivity properties prepared via peroxo-titanium complex catalyzed emulsion polymerization approach. Appl. Surf. Sci..

